# Secretome analysis revealed that cell wall remodeling and starch catabolism underlie the early stages of somatic embryogenesis in *Pinus nigra*

**DOI:** 10.3389/fpls.2023.1225424

**Published:** 2023-08-04

**Authors:** Miroslav Pernis, Terézia Salaj, Jana Bellová, Maksym Danchenko, Peter Baráth, Katarína Klubicová

**Affiliations:** ^1^ Institute of Plant Genetics and Biotechnology, Plant Science and Biodiversity Center, Slovak Academy of Sciences, Nitra, Slovakia; ^2^ Institute of Chemistry, Slovak Academy of Sciences, Bratislava, Slovakia

**Keywords:** extracellular proteome, peroxidases, contrasting embryogenic capacity, amylase, polygalacturonases, black pine

## Abstract

Somatic embryogenesis is an efficient mean for rapid micropropagation and preservation of the germplasm of valuable coniferous trees. Little is known about how the composition of secretome tracks down the level of embryogenic capacity. Unlike embryogenic tissue on solid medium, suspension cell cultures enable the study of extracellular proteins secreted into a liquid cultivation medium, avoiding contamination from destructured cells. Here, we present proteomic data of the secretome of *Pinus nigra* cell lines with contrasting embryogenic capacity, accounting for variability between genotypes. Our results showed that cell wall-related and carbohydrate-acting proteins were the most differentially accumulated. Peroxidases, extensin, α-amylase, plant basic secretory family protein (BSP), and basic secretory protease (S) were more abundant in the medium from the lines with high embryogenic capacity. In contrast, the medium from the low embryogenic capacity cell lines contained a higher amount of polygalacturonases, hothead protein, and expansin, which are generally associated with cell wall loosening or softening. These results corroborated the microscopic findings in cell lines with low embryogenic capacity—long suspensor cells without proper assembly. Furthermore, proteomic data were subsequently validated by peroxidase and α-amylase activity assays, and hence, we conclude that both tested enzyme activities can be considered potential markers of high embryogenic capacity.

## 1 Introduction

Somatic embryogenesis (SE) is a complex process by which somatic embryos develop from somatic cells under *in vitro* conditions. The process has been initiated in many plant species such as herbal species, angiosperm, and conifer trees. The developmental pattern of SE differs from that of angiosperms (Nagmani et al., 1995; Von Arnold et al., 2002). Von Arnold and Hakman (1988) distinguished four stages in the SE process: stage 1—bipolar somatic embryos formed by small dense meristematic cells and long vacuolated suspensor cells, stage 2—suspensor still attached to the more prominent meristematic cells, stage 3—appearance of cotyledons, and stage 4—green plantlets. The SE process sometimes occurs directly and sometimes indirectly through callus formation. Embryogenic tissues and non-embryogenic calli differ in morphology and color. Tissues are usually maintained on a solid medium. However, it is practical and convenient to establish suspension cultures for large-scale propagation. Single cells and cell aggregates directly contact the liquid culture medium in suspension cultures. As a result, the proliferation rate is higher, the cultures become synchronized, and they have a uniform metabolic state (Von Arnold et al., 2002; Miernyk et al., 2016). Cell suspension cultures are suitable for studying the role of secreted proteins during early plant development. Secreted proteins can be easily isolated from the cultivation medium, avoiding contamination by cytosolic proteins from disrupted cells (Cho et al., 2009; Gupta et al., 2011).

Secreted proteins are involved in the regulation of many biological and physiological processes, such as cell expansion, cell division, cell-to-cell communication, cell adhesion, signal transduction, and response to stress or stimuli (reviewed by Agrawal et al., 2010; Krause et al., 2013; Guerra-Guimarães et al., 2016). The relationship between the morphology of the somatic embryos and the secreted proteins was reported previously (Egertsdotter et al., 1993; Domon et al., 2000; Tchorbadjieva et al., 2004). Domon et al. (1995) described three extracellular glycoproteins specific for embryogenic cell lines of Caribbean pine (*Pinus caribaea* var. *hondurensis*). Hoenemann et al. (2010) studied SE by comparing transcriptomes of embryogenic and non-embryogenic calli of cyclamen. Their findings supported the idea that apoplast proteins play vital roles during SE. Besides these studies, further systematic analyses of secretomes comparing cell lines under differential developmental programs are missing.

Proteomic methods allow the large-scale analysis of protein dynamics during development. Focusing study on the subproteome level allows obtaining relevant information about the cell compartment as well as facilitates analysis of low abundant proteins. Proteomic studies of extracellular proteins based on the two-dimensional gel electrophoresis or protein prefractionation through one-dimensional gel followed by quantitative mass spectrometry suggested the role of α-amylases, different types of proteases, and glycoside hydrolases (xyloglucan endotransglucosylase/hydrolase, chitinases and glucanases) in the SE (Tchorbadjieva, 2016; Alves et al., 2017). Previously, we studied the SE of black pine (*Pinus nigra*) at the level of the cellular proteome. A comparison of three types of tissues obtained from two cell lines (embryogenic tissue with high regeneration capacity, non-embryogenic callus of the same genotype, and embryogenic tissue after loss of embryogenic capacity) revealed a higher impact of cell line/genotype on proteome than the type of investigated tissue. An altered accumulation of proteins active in the biosynthesis of cell wall components was also found, comparing the embryogenic and non-embryogenic tissue of both cell lines (Klubicová et al., 2017).

In the present study, we aimed to obtain comprehensive secretome profiles and to identify possible players in the SE of conifer *P. nigra*. We compared the population of proteins secreted into the suspension culture medium from cell lines with different embryogenic capacities by label-free mass spectrometry. In our comparison, we included two cell lines with high embryogenic capacity (HEC) and two with low embryogenic capacity (LEC) to weigh the influence of genotype. Our results contribute to a better understanding of complex regulation of early stages of SE, when bipolar structures are already formed. Finally, we proposed potential markers of early stages of SE in pine species.

## 2 Materials and methods

### 2.1 Tissue culture and induction of embryogenic cell lines

The embryogenic tissues were initiated from immature zygotic embryos of *P. nigra* enclosed in megagametophytes (details in Salaj et al., 2014). The initiated tissues were cultured on a basal DCR medium (Gupta and Durzan, 1985) containing plant growth regulators as BA (2.2 µM) and 2,4-D (9 µM), enzymatic casein hydrolysate (500 mg/L), glutamine (50 mg/L), myo-inositol (200 mg/L), glycine (2 mg/L), nicotinic acid (0.5 mg/L), pyridoxine (1 mg/L), and thiamine (1 mg/L). The medium was supplemented with 2% sucrose and solidified with 3% gelrite. For proliferation and long-term maintenance, the cultures were growing in clumps, kept in a growth chamber in the dark at 23°C, and subcultured in 2-week intervals. Cell lines E489, E490, E492, and E494 were used for secretome analysis; cell lines E489, E490, E483, E482, E486, and E494 were used for peroxidase activity assay; and cell lines E489, E490, E483, E456, E477, and E494 were used for α-amylase activity assay.

### 2.2 Microscopic observation

The structural features of early somatic embryos were followed using squash preparations. Small pieces of well-growing tissue were separated from clumps, placed on a glass slide, stained with acetocarmine (2% w/v), squashed, and covered by a coverslip (Salajová and Salaj, 2005). The preparations were investigated under an Axioplan 2 microscope (Carl Zeiss Microscopy, LLC) equipped with a camera system DXC-5500 (Sony).

### 2.3 Somatic embryo maturation

On the eighth to ninth day after the last subculture, the tissues were resuspended in a liquid basal DCR medium without growth regulators and organic additives. Aliquots of 100–120 mg of tissue in the suspension were pipetted on stacked filter paper discs, and after absorbing the liquid, the filter papers with cells on their surface were placed on a maturation medium. The maturation medium was based on basal DCR medium supplemented with ABA (95 µM, Sigma), enzymatic casein hydrolysate (500 mg/L), glutamine (50 mg/L), myo-inositol (200 mg/L), glycine (2 mg/L), nicotinic acid (0.5 mg/L), pyridoxine (1 mg/L), thiamine (1 mg/L), and maltose 6%. The maturation medium was solidified with 1% gelrite (details in Salaj et al., 2014). After approximately 5 weeks of culture, the number of precotyledonary somatic embryos was calculated, and later, when cotyledonary somatic embryos appeared (approximately after 8 weeks on maturation medium), their number was also recorded. The embryogenic capacity was evaluated in five replicates and was expressed as a number of somatic embryos calculated per 1 g of fresh mass.

### 2.4 Suspension culture preparation and sample collection

Four cell lines were selected for secretome analysis based on their microscopic characterization and embryogenic capacity: E489, E490 (LEC) and E492, E494 (HEC). On the eighth day after the last subculture, 2.5 g of well-growing embryogenic tissue (taken from different plates) was resuspended in 25 ml of liquid basal DCR proliferation medium. The suspension cultures were cultivated in 100-ml Erlenmeyer flasks on a rotary shaker at 80–90 rpm in the dark. After 1 week of cultivation, the suspension was poured into 25-ml glass cylinders, and the cells were allowed to settle for 30 min. We estimated the sedimented cell volume (SCV; ml) as a non-destructive growth parameter (Find et al., 1998; Salaj et al., 2007). Subsequently, 3 ml of SCV was pipetted to 22 ml of fresh liquid proliferation medium to obtain the final volume of 25 ml. The suspensions were cultured as described above for the following 10 days. After 10 days, the SCV was estimated again (ml), and the culture medium was filtered using a sterile syringe filter with a 0.2-µm pore size (Sarstedt). Culture medium with the addition of cOmplete EDTA-free protease inhibitor (Roche) was stored at −80 °C until further analysis.

### 2.5 Protein extraction and quality control

Extraction of secreted proteins was performed in duplicate for each cell line. The culture medium (50 ml) was concentrated and partially desalted in two steps by ultrafiltration at 4 °C using Amicon Ultra-15 (10 kDa cutoff, Millipore) and Amicon Ultra-0.5 (3 kDa cutoff, Millipore) to a final volume of 200 µl. According to the manufacturer’s instructions, concentrated secreted proteins were precipitated with 2-D Clean-Up Kit (GE Healthcare), and pellets were solubilized in 40 µl of solubilization buffer (4% SDS and 120 mM Tris-HCl, pH 6.8). Proteins were quantified using the Pierce BCA Protein Assay kit (Thermo Scientific).

To estimate potential contamination by cytosolic proteins, the activity assay of cytosolic enzyme glucose-6-phosphate dehydrogenase (G6PDH; EC 1.1.1.49) was performed using the G6PDH assay kit (Sigma-Aldrich) according to the manufacturer’s instructions.

Secreted proteins were separated in 12% polyacrylamide separation gel with 4% upper stacking gel. Prior to loading, protein samples (25 µg) were mixed with loading buffer (200 mM DTT, 20% glycerol, 0.1% bromophenol blue) in a 1:1 ratio and heated at 70 °C for 10 min. Electrophoresis was performed on the Protean II xi Cell unit (gel dimensions 16 × 20 cm) (Bio-Rad) as follows: constant voltage of 60 V (30 V per gel) until the dye front reached separation gel, then at a constant voltage of 180 V (90 V per gel) until the run was complete. The protein bands were visualized with colloidal Coomassie blue (G-250) and digitalized using the GS-800 Calibrated Densitometer (Bio-Rad).

### 2.6 In-gel digestion of secreted proteins

Each gel lane was divided into nine sections of similar size and cut into small pieces. Gel pieces were washed with 50 mM ammonium bicarbonate containing 50% acetonitrile until complete removal of the dye. Subsequently, gel pieces were reduced with 10 mM dithiothreitol in 100 mM ammonium bicarbonate, alkylated with 50 mM iodoacetamide in 100 mM ammonium bicarbonate, and dehydrated with 100% acetonitrile. Finally, the gel pieces were saturated with 10 ng/µl trypsin (Promega) in 10 mM ammonium bicarbonate containing 10% acetonitrile for 2 h at 4 °C and digested at 37 °C overnight. The digestion was stopped by 1% trifluoroacetic acid in 70% acetonitrile and tryptic peptides were extracted by 70% acetonitrile with 1% trifluoroacetic acid. Extracted peptides were stored at −20 °C until analysis.

### 2.7 Protein identification and quantification by mass spectrometry

Extracted peptides from each sample lane were pooled, dried, and dissolved in 0.1% TFA and 2% acetonitrile (ACN). The samples were loaded onto a trap column (Acclaim PepMap100 C18, 75 µm × 20 mm, Dionex) and separated with a C18 column (Acclaim PepMap C18, 75 µm × 150 mm, Dionex) on an Ultimate 3000 RSLCnano system (Dionex) in a linear 120-min gradient (3%–43% B) and a flow rate of 300 nl/min. The following two mobile phases were used: 0.1% FA (v/v) (A) and 80% ACN (v/v) with 0.1% FA (B). Eluted peptides were sprayed directly into an Orbitrap Elite mass spectrometer (ThermoScientific, MA, USA) and spectral datasets were collected in the data-dependent mode using the Top 15 strategy for the selection of precursor ions for the HCD fragmentation (Michalski et al., 2012). Each of the samples was analyzed in two technical replicates. Obtained datasets were processed by MaxQuant (version 1.5.3.30, Cox and Mann, 2008) with a built-in Andromeda search engine using carbamidomethylation (C) as permanent and oxidation (M) as variable modifications. A label-free quantitation algorithm was used to determine relative protein quantities. The search was performed against the *Pinus taeda* protein database (Romero-Rodríguez et al., 2014). The mass spectrometry proteomics data have been deposited to the ProteomeXchange Consortium via the PRIDE partner repository with dataset identifier PXD040247 available at https://www.ebi.ac.uk/pride/archive/projects/PXD040247 (Perez-Riverol et al., 2022).

### 2.8 Enzyme activities

Peroxidase activity was measured in the cell culture medium of cell lines E489, E490, and E483 (LEC) and E494, E482, and E486 (HEC) in triplicate. The secreted proteins for the peroxidase activity assay were concentrated as described above, followed by buffer exchange [phosphate-buffered saline (PBS; 0.01 M, pH 7.4)] after the ultrafiltration step. Peroxidase activity was determined spectrophotometrically at 420 nm using the Peroxidase (POD) Assay kit (Elabscience) according to the manufacturer’s instructions. Peroxidase activity was expressed in units per milligram of secreted protein (U/mg protein). One unit was defined as the enzyme activity that catalyzes 1 µg of substrate by the 1 mg of protein extract per minute at 37 °C.

The α-amylase activity was measured in the culture medium of cell lines E489, E490, and E483 (LEC) and E456, E477, and E494 (HEC) in triplicate. Secreted proteins were concentrated using Amicon Ultra-15 (10 kDa cutoff, Millipore). The activity was determined spectrophotometrically at 405 nm using the Amylase Activity Assay Kit (Sigma Aldrich) according to the manufacturer’s instructions. The α-amylase activity was expressed in nmol/min·ml. One unit is the amount of α-amylase that cleaves ethylidene-pNP-g7 to generate 1.0 μmol of p-nitrophenol per minute at 25 °C.

### 2.9 Statistics and bioinformatic analysis

A powerful classification approach, partial least square discriminant analysis (PLS-DA) with pareto scaling, was carried out by script EZinfo 3 for Waters (Umetrics). In essence, PLS-DA discovers a combination of variables (specific proteins), which separate sample groups (cell lines with HEC versus cell lines with LEC). To assess the relative influence of a proteomic variable on the mathematical model, variable importance in the projection (VIP) scores were calculated. VIP score is a weighted sum of the squared correlations between elements of the model. Additionally, conventional statistics (Student’s *t*-test) on proteomic data was done in Perseus (version 1.5.5.3). Two cell lines with similar embryogenic capacity were treated as replicates. Statistical evaluation of enzymatic activity essays was done in Prism (version 9.3, GraphPad) using ANOVA followed by *post-hoc* Tukey test. Each of the six cell lines was considered as a separate group.

To predict the subcellular localization of differentially accumulated proteins, we used a set of bioinformatic tools. The presence of signal peptides (SP) was predicted using SignalP 5.0 server (http://www.cbs.dtu.dk/services/SignalP-5.0/index.php) and TargetP-2.0 server (https://services.healthtech.dtu.dk/services/TargetP-2.0/). Only proteins predicted to have a signal peptide by both programs were considered. Those proteins were further scanned against PS00014 motif using the ScanProsite server (https://prosite.expasy.org/scanprosite/) to determine if they are retained in the endoplasmic reticulum (ER). Proteins containing ER retention motif were excluded from the list of secreted proteins. The localization prediction was accepted when three out of four used prediction tools [WegoLoc (http://www.btool.org/wegoloc), PredSL (http://aias.biol.uoa.gr/PredSL/index.html), Euloc (http://euloc.mbc.nctu.edu.tw/index.html), and Fuel-mLoc (http://bioinfo.eie.polyu.edu.hk/FUEL-mLoc/)] concurred.

The PredGPI prediction tool (http://gpcr2.biocomp.unibo.it/gpipe/index.htm) was used for the prediction of GPI-anchored proteins (specificity: ≥99.5%). For the prediction of transmembrane helices in proteins, they were queried in TMHMM Server v. 2.0 (http://www.cbs.dtu.dk/services/TMHMM/), CCTOP (http://cctop.enzim.ttk.mta.hu/), and MEMSAT analysis tool on PSIPRED server (http://bioinf.cs.ucl.ac.uk/psipred/). Transmembrane predictions were accepted in the case of the presence in at least two predictors. We assigned the most probable location for each identified protein by cross-referencing data obtained from the bioinformatics tools mentioned above.

## 3 Results

### 3.1 Characterization of the embryogenic capacity of analyzed cell lines

**Figure 1:**
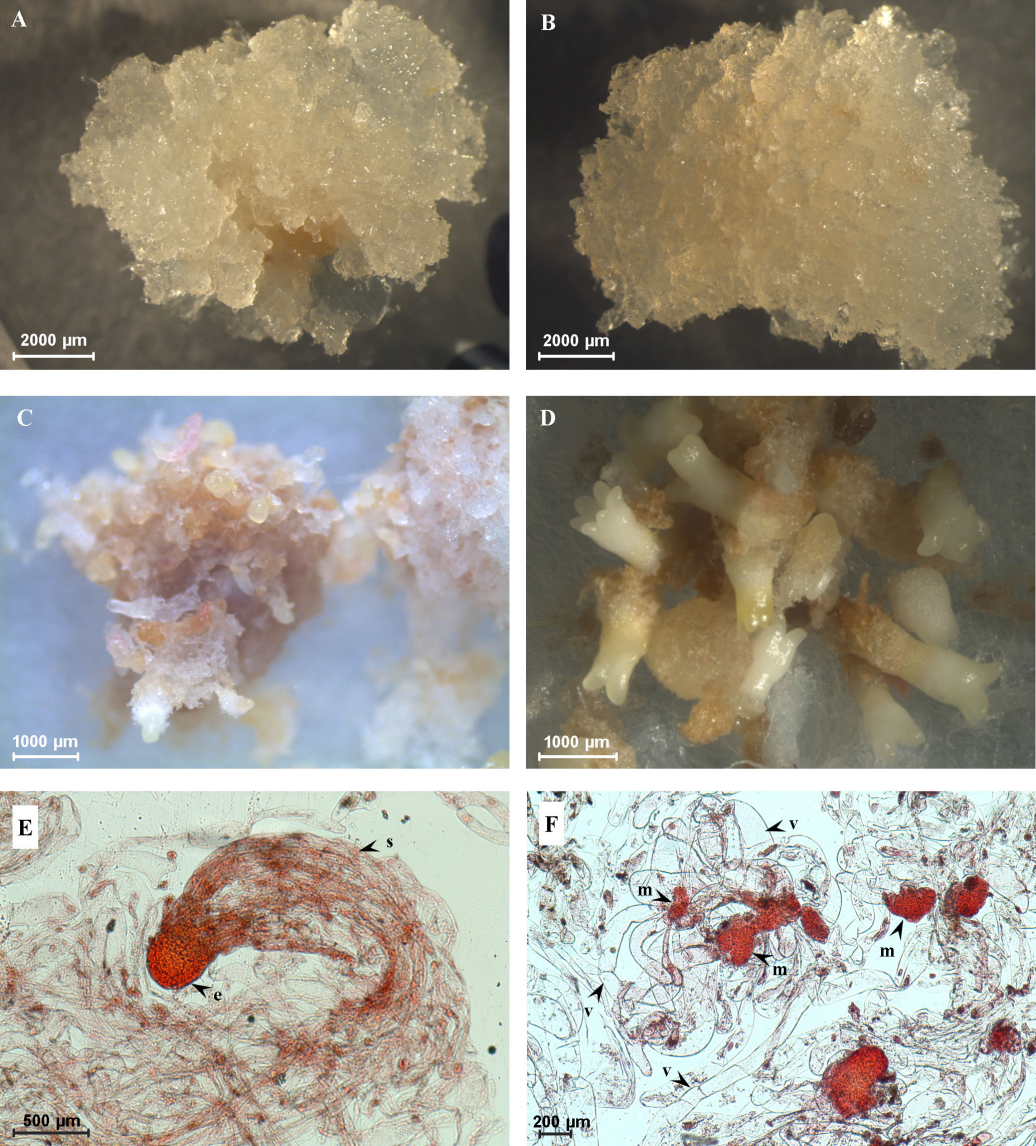
Characterization of cell lines with high embryogenic capacity (HEC) and low embryogenic capacity (LEC). **(A)** HEC tissue on the proliferation medium. **(B)** LEC tissue on the proliferation medium. **(C)** Precotyledonary somatic embryos in HEC cell lines after 5 weeks of development on the maturation medium. **(D)** Cotyledonary somatic embryos in HEC cell lines after 8 weeks on maturation medium. **(E)** Bipolar somatic embryo observed in HEC tissue growing on the proliferation medium (e, embryonal part composed of tightly packed meristematic cells; s, suspensor composed of a bundle of long vacuolated cells). **(F)** Loosely organized meristematic cells (m) connected with long vacuolated suspensor cells without assembly into the bundle (v) observed in LEC tissue growing on the proliferation medium.

A total of nine embryogenic cell lines were selected for the analyses based on embryogenic capacity, i.e., based on numbers of formed cotyledonary somatic embryos. Six cell lines (E456, E477, E482, E486, E492, and E494) were classified as HEC cell lines (**Figure 1A**) and cell lines E483, E489, and E490 represented LEC cell lines (**Figure 1B**). In HEC cell lines, after approximately 5 weeks of cultivation on the maturation medium, precotyledonary somatic embryos (**Figure 1C**) started to appear in high numbers (456–2,354 precotyledonary somatic embryos per gram of fresh weight), and on average, 30%–35% of these structures (199–690 cotyledonary somatic embryos per gram of fresh weight) developed further, reaching the cotyledonary developmental stage (**Figure 1D**; **Supplementary Table 1**). Their development terminated in somatic seedlings regeneration.

Precotyledonary somatic embryos appeared in LEC cell lines as well, but in much lower numbers (72–111 precotyledonary somatic embryos per gram of fresh weight); moreover, they did not reach the cotyledonary developmental stage. These structures necrotized or were overgrown due to intensive tissue cell proliferation. Two types of cell lines also differed in the structural organization of early stages of bipolar somatic embryos. HEC cell lines contained bipolar somatic embryos with well-formed embryonal parts composed of tightly packed meristematic cells (“head”). The embryonal part was connected with a suspensor composed of a bundle of long vacuolated cells (**Figure 1E**). In this stage, shoot and root apical meristems are not differentiated. In LEC cell lines, the embryonal meristematic cells are organized as loosely packed aggregates connected with long vacuolated suspensor cells without assembly into the bundle (**Figure 1F**).

### 3.2 Global overview of secretome profile

We used culture media to study secreted proteins with a potential role in the early stages of SE. We took into proteomic analysis four cell lines of *P. nigra* with different regeneration capacity: two LEC (E489 and E490) and two HEC cell lines (E492 and E494) growing in the proliferation basal DCR medium. We analyzed secreted proteins by the label-free LC-MS/MS approach with quality control of protein extract by denaturing gel electrophoresis in two biological replicates per cell line (**Supplementary Figure 1**). Only proteins with at least two identified peptides were considered for analysis. This approach resulted in the identification and quantification of 187 protein accessions in total (**Supplementary Table 2**). Among them, 42 proteins were detected in significantly altered amount when comparing two types of embryogenic tissues. Many of them are products of complex gene families with multiple functions. Functional classification of these proteins revealed that most represented groups are enzymes acting on cell wall carbohydrates (15). Proteins with miscellaneous function (8), proteases (7), and oxidoreductases (5) are highly represented as well. Finally, proteins active in lipid metabolism (2), nucleic acid metabolism (2), carbohydrate metabolism (1), signal transduction (1), and structural protein (1) were found as well (**Table 1**). Specific information about each differentially abundant protein is described together with discussion of its possible implication in SE in *Section 4*.

**Table 1:** Functional classification of 42 proteins secreted into the culture media from cell lines with high and low embryogenic capacity in altered amount.

Protein name	Accession	*p*-value	Fold change	Subcellular localization prediction	UniProt/NCBI Blast	
					E-value	Identity
Proteins acting on cell wall carbohydrates						
Alpha-xylosidase 1	PITA_000040514	0.012	−3.3	Extracellular	0.0	55%
Beta-xylosidase/alpha-L-arabinofuranosidase 2-like	PITA_000020490	0.028	1.7	Extracellular	–	–
Cellulase domain-containing protein	PITA_000028541	0.001	−2.4	Extracellular	8.9e-73	61%
Cellulase protein isoform 2	PITA_000095139	0.025	−1.2	Extracellular	8.1e-113	63%
Glucan endo-1,3-alpha-glucosidase agn1	PITA_000019465	0.019	−0.9	Extracellular, cell wall	2E-71*	50%*
Putative glycoside hydrolase family 17	PITA_000076475	9.16E-05	−2.8	Extracellular	5.7e-139	42%
Glycoside hydrolase	PITA_000023687	0.000	−1.4	Extracellular (GPI anchor)	0.0	68%
(1->3)-Beta-glucan endohydrolase	PITA_000070350	0.009	1.7	Extracellular	–	–
Glucan endo-13-beta-glucosidase 13	PITA_000082290	0.030	1.9	Extracellular	1.5e-55	41%
Xyloglucan endotransglucosylase/hydrolase	PITA_000065156	0.038	−1.3	Extracellular	–	–
Xyloglucan endotransglucosylase/hydrolase	PITA_000008828	0.032	2.1	Extracellular	9.4e-102	56%
Polygalacturonase/glycoside hydrolase family protein	PITA_000022582	0.031	1.7	Pl. membrane	0.0	67%
Polygalacturonase/glycoside hydrolase family protein	PITA_000023506	0.018	2.1	Pl. membrane	0.0	66%
Heparanase-like protein 3	PITA_000022045	0.012	2.6	Extracellular	–	–
Expansin-like A1	PITA_000096460	0.026	4.3	Extracellular	**-**	**-**
Oxido-reductases						
Peroxidase, heme binding, substrate b., active b. calcium b	PITA_000058413	0.006	−2.9	Extracellular	**-**	**-**
Peroxidase, heme binding, substrate b., active b. calcium b.	PITA_000072979	0.0004	−2.5	Extracellular	1.7e-152	64%
Peroxidase, heme binding, substrate b., active b. calcium b	PITA_000047715	0.011	−1.6	Extracellular	–	–
Peroxidase (class III) substrate binding	PITA_000021812	0.043	−1.4	Extracellular	–	–
Protein HOTHEAD	PITA_000090479	0.021	1.3	Extracellular	–	–
Carbohydrate metabolism						
Alpha-amylase	PITA_000022208	0.004	−2.7	Extracellular	1.7e-179	58%
Nucleic acid metabolism						
Endoribonuclease L-PSP family protein	PITA_000036322	0.005	1.8	Pl. membrane	–	–
Nuclease S1	PITA_000001910	0.009	3.2	Extracellular	**-**	**-**
Signal transduction						
LysM-domain containing receptor-like kinase	PITA_000007354	0.012	−0.7	Pl. membrane	–	–
Lipid metabolism						
GDSL esterase/lipase	PITA_000012241	0.037	−1.6	Extracellular	–	–
Glycerophosphodiester phosphodiesterase GDPDL4-like	PITA_000061271	0.045	0.4	Pl. membrane	4E-97*	46.57%*
Proteases						
Basic secretory protease (S)	PITA_000055798	0.002	−2.2	Extracellular	1.4e-90	62%
Plant basic secretory family protein (BSP)	PITA_000007634	0.019	−2.1	Extracellular	8.3e-144	52%
Low-temperature-induced cysteine proteinase	PITA_000024486	0.018	1.04	Extracellular	0.0	70%
Cysteine proteinase RD21A-like	PITA_000024485	0.008	1.1	Extracellular	0.0	71%
Peptide-N4-(N-acetyl-beta-glucosaminyl) asparagine amidase A	PITA_000037874	0.001	1.6	Extracellular, pl. membrane	–	–
Carboxypeptidase	PITA_000006673	0.004	1.8	Extracellular	3.8e-172	53%
Carboxypeptidase	PITA_000006675	0.029	2.4	Extracellular	7.5e-54	65%
Miscellaneous proteins						
Pathogenesis-related thaumatin-like protein 3.3	PITA_000000597	0.006	−1.8	Extracellular	5.9e-109	70%
Thaumatin-like protein	PITA_000033081	0.050	−1.3	Extracellular	3.1e-74	59%
Pathogenesis-related thaumatin-like protein 3.3	PITA_000010735	0.040	−1.3	Extracellular	2.5e-119	71%
Antifungal protein ginkbilobin-like protein	PITA_000094670	0.020	−1.7	Extracellular	2.6e-48	92%
Ginkbilobin 2 (Gnk2) domain protein	PITA_000015772	0.039	−1.8	Pl. membrane	–	–
Phytocyanin domain-containing protein	PITA_000084523	0.041	−1.3	Pl. membrane (GPI anchor)	2.8e-65	44%
Early nodulin-like protein 14	PITA_000005806	0.006	3.1	extracellular (GPI anchor)	2E-34*	54%*
Putative dirigent protein 9.21	PITA_000087848	0.024	-1.0	Extracellular	–	–
Structural proteins						
Cell wall hydroxyproline-rich glycoprotein-extensin	PITA_000048168	0.040	−1.7	Extracellular	–	–

The table shows protein name, accession, p-value, fold change, subcellular localization prediction, and UniProt/NCBI Blast—E-value and identity after BLAST in the UniProt (https://www.uniprot.org/) or NCBI (https://www.ncbi.nlm.nih.gov/) database.*NCBI Blast, pl.,3 plasma.

**Figure 2:**
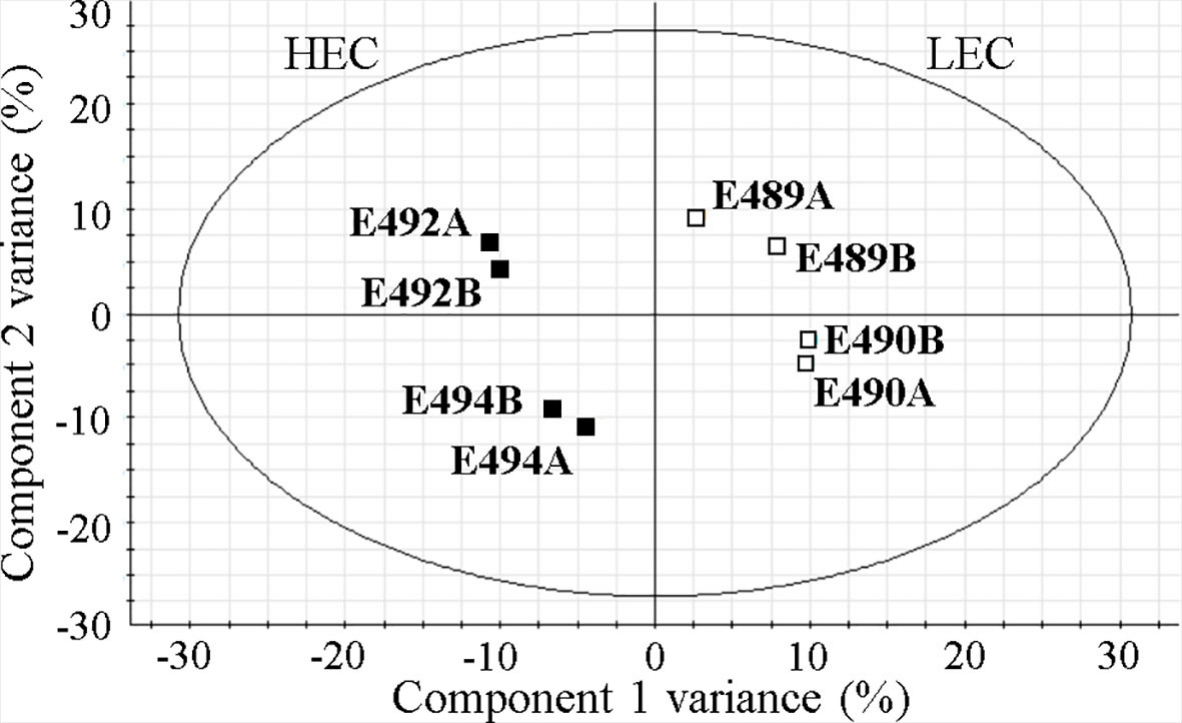
Visualization of the mathematical model in partial least square discriminant analysis (PLS-DA) of quantified proteins. HEC, high embryogenic capacity cell lines; LEC, low embryogenic capacity cell lines; A and B, biological replicates of a specific cell line.

The principal component analysis showed the expected clustering of experimental replicates in two-dimensional space, ensuring the reliability of findings. Multidimensional statistics PLS-DA confirmed that differentially accumulated proteins substantially contributed (VIP scores > 1.5) to the separation of biological groups in the mathematical model (**Figure 2**). The most highly rated proteins with VIP scores > 1.8 were as follows: expansin-like A1, α-xylosidase 1, putative glycoside hydrolase family 17, early nodulin-like protein 14, nuclease S1, two peroxidase isoforms (PITA_000058413 and PITA_000072979), α-amylase, and cellulase domain-containing protein.

### 3.3 Enzyme activities

**Figure 3:**
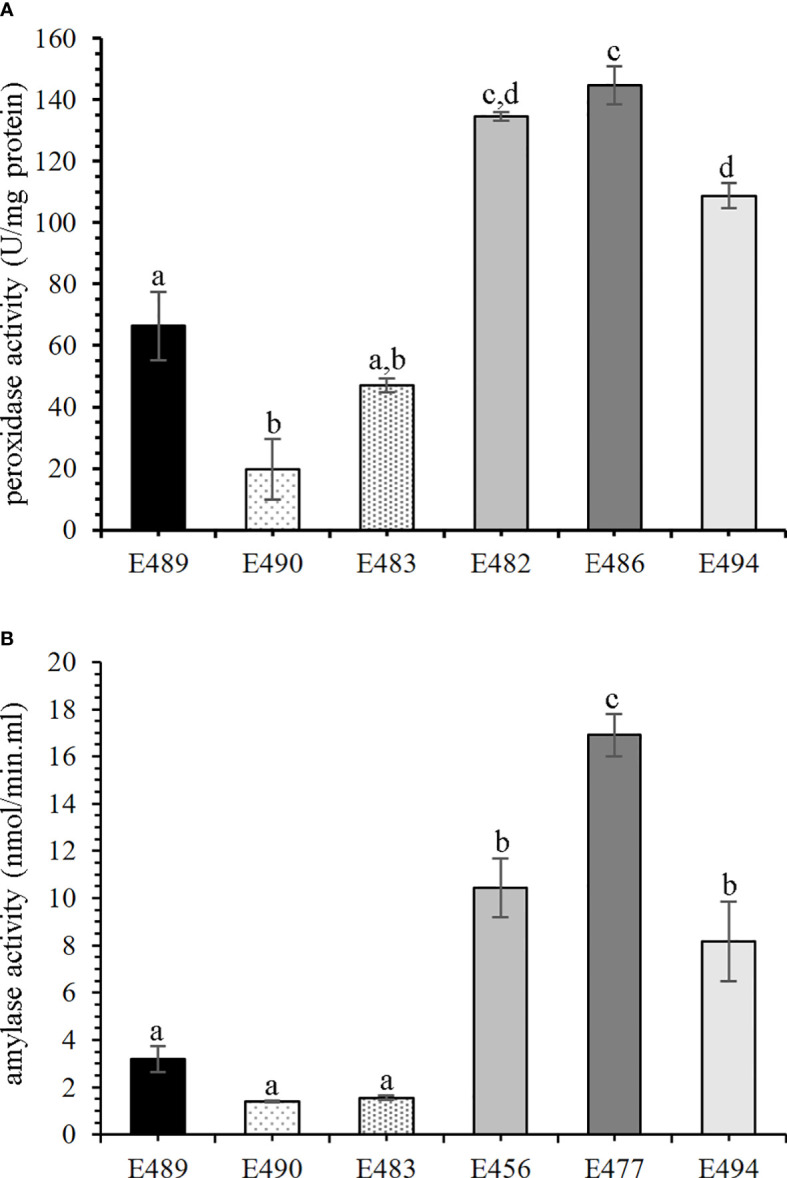
Enzyme activities in culture media of cell lines with different embryogenic capacity. Mean values with the same letters are not significantly different according to Tukey’s test, *p* < 0.05, *n* = 3. Error bars indicate the standard error of the mean. Statistical analysis revealed a significant difference in enzyme activities between the low embryogenic capacity cell line (LEC) and high embryogenic capacity cell line (HEC) groups. **(A)** Peroxidase (POD) activity; LEC: E489, E490, and E483; HEC: E482, E486, and E494. **(B)** α-Amylase activity; LEC: E489, E490, and E483; HEC: E456, E477, and E494.

In order to verify if altered accumulation of enzymes correlated with the activity in the culture medium, we performed standardized peroxidase activity assay and α-amylase activity assay. We performed the G6PDH enzyme activity test to estimate potential contamination by cytosolic proteins, and we did not detect any activity. To confirm if the peroxidase activity in the medium could serve as a marker of embryogenic capacity during early stages of SE in *P. nigra*, we also included medium from additional cell lines into the analysis. Altogether, peroxidase activity was measured in the medium from six cell lines: E489, E490, and E483 (LEC), and E482, E486, and E494 (HEC). Detected peroxidase activity was in the range 19.7 ± 9.8 to 66.4 ± 11 U/mg protein in medium from LEC cell lines, and differentially higher activity (108.8 ± 4.1 to 144.8 ± 6.1 U/mg protein) was measured in the medium from HEC cell lines (**Figure 3A**). The α-amylase activity was measured in culture medium from six cell lines—E489, E490, and E483 (LEC), and E456, E477, and E494 (HEC)—and was statistically higher in the medium from HEC cell lines (8.2 ± 1.7 to 16.9 ± 0.9 nmol/min·ml) than in the medium from LEC cell lines (1.4 ± 0.04 to 3.19 ± 0.5 nmol/min·ml; **Figure 3B**).

## 4 Discussion

### 4.1 Differential abundance of cell wall-related proteins in embryogenic lines

The cell wall is a complex and dynamic structure that consists of various components such as polysaccharides, lignin, and proteins and plays an essential role in controlling the size and shape of the plant cell. The modification of the cell wall composition is required during cell division, cell elongation, and differentiation, which occur in response to environmental stress or during development. Therefore it is not surprising that most proteins with altered abundance are related to cell wall modifications, thus affecting cell-to-cell communications, which is important for proper embryo development.

**Figure 4:**
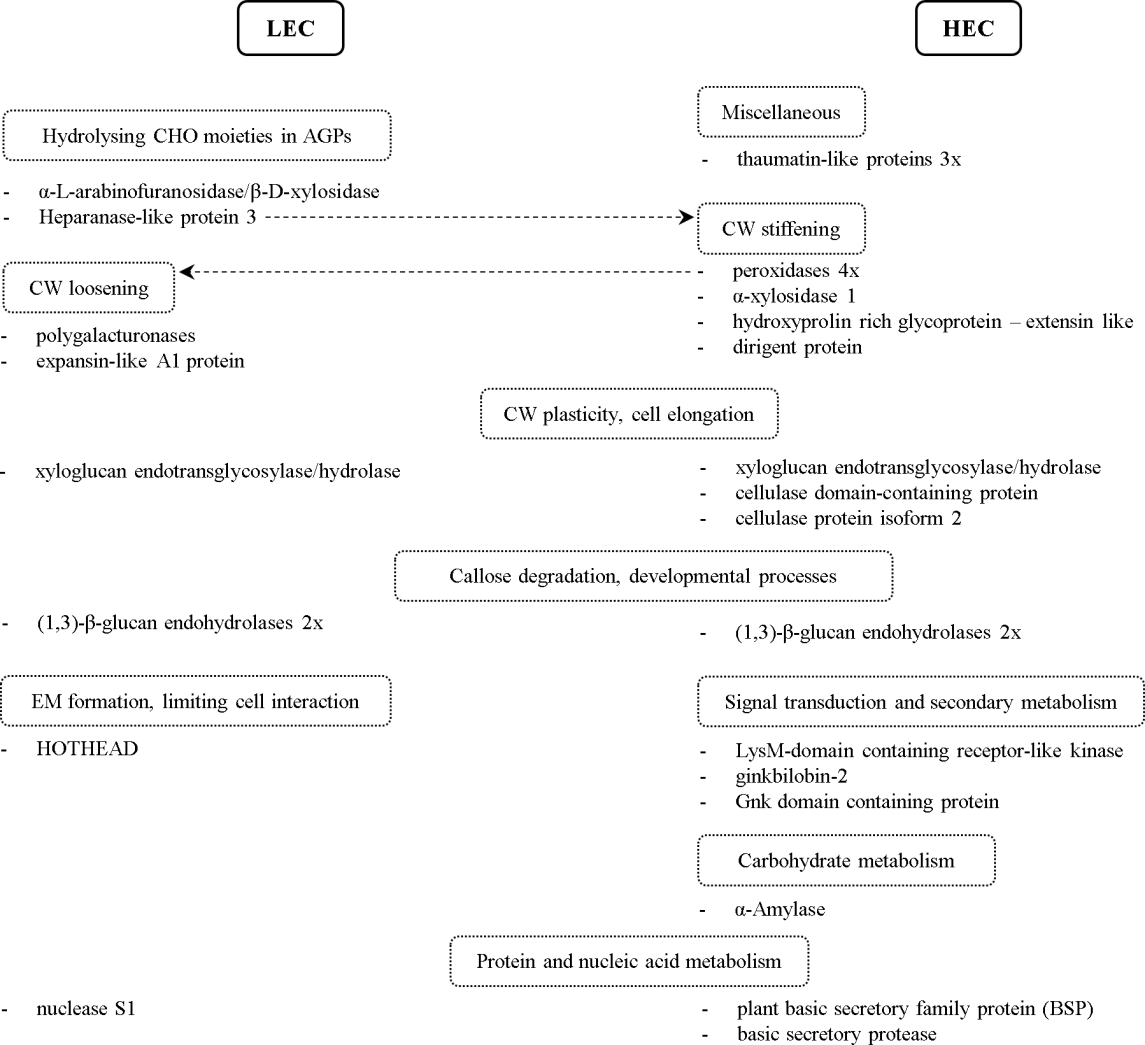
Schematic overview of proteins with altered accumulation in comparison between cell lines and their classification into groups by their biological function. The dashed line points to an alternative protein function. LEC, low embryogenic capacity cell lines; HEC, high embryogenic capacity cell lines; CHO, carbohydrates; AGPs, arabinogalactan proteins; CW, cell wall; EM, extracellular matrix.

Our comparative approach revealed a differential accumulation of cell wall remodeling proteins. Most of them are members of several glycoside hydrolase superfamilies (GH) acting on the different components of the cell wall, and their activity is often affected by post-translational modifications (Minic and Jouanin, 2006; Chandrasekar et al., 2014). Among those, an α-xylosidase 1 was more abundant in the medium from HEC cell lines (**Table 1**; **Figure 4**). This enzyme is involved in the modification of xyloglucan structure and thus influences cell wall integrity (Shigeyama et al., 2016) and is required to maintain the physical strength of the primary cell wall in growing tissues. α-Xylosidase 1 was found in the proteome during the early stages of zygotic embryo development and under conditions necessary for proper somatic embryo development (Balbuena et al., 2009; Morel et al., 2014). Other GHs accumulated in the medium from HEC cell lines are cellulase domain-containing protein and cellulase protein isoform 2 (**Table 1**; **Figure 4**). Cellulases belong to the GH9 family and act on a broad range of substrates (Yoshida and Komae, 2006). Cellulase genes were expressed in rapidly growing and differentiating reproductive structures and in vegetative tissues where cell expansion occurs, including the embryogenic tissue of *Pinus radiata* (Loopstra et al., 1998; Bishop-Hurley et al., 2003; Hartati et al., 2008). However, overexpression of the cellulase gene did not result in a decreased level of cellulose, but rather caused a decrease of xyloglucan cross-linked with cellulose microfibrils, thus allowing increased cell wall plasticity (Park et al., 2003). Cell wall plasticity affects cell division, cell elongation, cell shape, and, thus, proper somatic embryo development.

Among GH more abundant in the medium from LEC cell lines, we found heparanase-like protein 3, β-D-xylosidase/α-L-arabinofuranosidase, and two polygalacturonases (**Table 1**; **Figure 4**). Polygalacturonases are multigene family proteins driving pectin degradation. Constitutive overexpression of polygalacturonase in the apple led to the reduction of pectin as a major component of the cell wall and abnormal cell separation within the leaf (Atkinson et al., 2002). For LEC cell lines, it is typical that the embryonal part of somatic embryos (**Figure 1F**) consists of meristematic cells that are only loosely aggregated and attached to the long vacuolated suspensor cells, which are not organized into the bundles (Salajová and Salaj, 2005). Similarly, the much looser cell adhesion was observed in the non-embryogenic cell line of *Cyclamen persicum*, and it was proposed as a potential morphological marker of SE (Hoenemann et al., 2010).

Plant heparanases belong to the GH79 family and have β-glucuronidase (GUS) activity. The β-glucuronidase indirectly participates in H_2_O_2_ degradation and generates phenolic compounds that may be used in cell wall fortification, thus affecting cell wall remodeling (Eckey et al., 2004; Belchí-Navarro et al., 2019). Eudes et al. (2008) suggested its role in the regulation of cell growth through hydrolyzing glucuronic acids from glycan chains of arabinogalactan proteins (AGPs). Another enzyme putatively hydrolyzing carbohydrate moieties of AGPs is β-D-xylosidase/α-L-arabinofuranosidase (Kotake et al., 2006). The authors observed its high expression in immature radish seeds and proposed its role in seed germination or embryogenesis. Their results indicate that it could be involved in post-translational modifications of glycoproteins or may participate in the degradation of AGPs (Kotake et al., 2006; Eudes et al., 2008). Several studies revealed that the glycan chains are essential for AGPs function during SE (Šamaj et al., 2008), cell–cell interaction (Motose et al., 2004), and cell aggregation (Capataz-Tafur et al., 2011).

Additionally, we identified two isoforms of xyloglucan endotransglycosylase/hydrolase (XTH; EC 2.4.1.207) of the GH16 family, each accumulated in the medium from different types of analyzed cell lines (**Table 1**; **Figure 4**). XTHs are apoplastic enzymes with both xyloglucan endotransglucosylase (XET) and xyloglucan endohydrolase (XEH) activities (Franková and Fry, 2013), which cleave and reassemble xyloglucan polymers and thus influence CW plasticity and cell elongation. Similarly, we detected different abundance of four glycoside hydrolases of GS17 family with homology to (1,3)-β-glucan endohydrolase (or β-1,3-glucanase), two of them more abundant in the medium from LEC and two in the medium from HEC cell lines. Besides a well-described role in stress response (Jin et al., 2007; Zieliński et al., 2021), they play a significant role in many developmental processes such as seed development and germination (Buchner et al., 2002; Leubner-Metzger, 2003) and cell division (Doxey et al., 2007). β-1,3-Glucanases may be involved in the degradation of the callose surrounding embryogenic structures and small embryos (Helleboid et al., 2000). From our data, it is not possible to conclude if the different isoforms of extracellular β-1,3-glucanases can complement each other or if their function is specific and, consequently, influence the embryogenic capacity. Several glycoside hydrolases, including β-1,3-glucanases and xyloglucan endotransglycosylase/hydrolase, were already suggested to play a role in SE (Helleboid et al., 1998; Malinowski et al., 2004; Balbuena et al., 2009; Vale et al., 2014).

We detected accumulation of four out of nine quantified peroxidase isoforms, belonging to Class III (EC 1.11.1.7), hydroxyproline-rich glycoprotein (HRGP) with homology to leucine-rich repeat extensin (EXT) proteins, and dirigent protein (DIR) in the medium from HEC cell lines (**Table 1**; **Figure 4**). Peroxidases are bifunctional enzymes that can catalyze the reduction or the release of H_2_O_2_ and reactive oxygen species, thus causing a wide range of cell wall modifications. As a result of their action, cell wall loosening or cell wall stiffening may occur. Cell wall stiffening through the formation of suberin, lignin, or cross-linking of cell wall components, such as extensins, led to the inhibition of cell elongation growth (Passardi et al., 2004; Mishler-Elmore et al., 2021). Overexpression of DIR genes resulted in increased lignin formation (Shi et al., 2012), and cell wall lignification was suggested to be a crucial step during SE (Li et al., 2023). On the other side, cell wall loosening is necessary for cell elongation (Liszkay et al., 2003; Passardi et al., 2004) and controlled cell elongation is essential for proper suspensor formation. Therefore, maintaining the balance and proper localization of cell wall loosening or stiffening is essential for the control of elongation cell growth during proper somatic embryo development. The role of peroxidases in the early stages of SE of several species was proposed based on their increased activity in the culture medium and cell wall fraction of spruce and asparagus (Mo et al., 1996; Takeda et al., 2003). Our results support the notion that the higher abundance of several isoforms of peroxidases is also reflected in the increased peroxidase activity in the culture medium, hence can serve as a marker of embryogenic capacity.

Three thaumatin-like proteins (TLPs), all more abundant in the medium from HEC cell lines (**Table 1**; **Figure 4**), were previously suggested to play a role in the pollination of gymnosperm (Coulter et al., 2012) and early stages of SE of *Quercus* (Gomez-Garay et al., 2013). TLPs affect plant cell wall by hydrolyzing β-1,3-glucans (Grenier et al., 1999). Increased thaumatin-like protein abundance correlated with increased peroxidase activity during water deficit (Pinheiro et al., 2001), similarly to our findings. Our observations, therefore, support the previous results indicating the role of TLPs not only as pathogenesis-related proteins but also in somatic embryo development.

α-Amylase (EC 3.2.1.1), more abundant in the medium from HEC cell lines (**Table 1**; **Figure 4**), participates in starch degradation (Stanley et al., 2002; Stanley et al., 2005). The accumulation of starch, as a vital storage compound in plants, depends on the balance between synthesis and degradation. Navarro et al. (2017) observed the relationship between sucrose and starch accumulation and the responsiveness of the embryogenic cell lines. Starch accumulation in the responsive cell line and zygotic embryogenesis of *Araucaria angustifolia* occurred at the later stages of development. Contrastingly, starch accumulated early in the proliferation phase in the blocked line, and starch degradation occurred during the maturation phase (Navarro et al., 2017). In accordance with these findings, higher abundance of α-amylase in the medium from HEC cell lines resulted in increased enzymatic activity in the culture medium and allowed us to distinguish cell lines based on the embryogenic capacity. Therefore, α-amylase activity in the culture medium can be considered as an additional potential marker of embryogenic capacity in *P. nigra*. Previously, it was also proposed as an early marker of SE of *Dactylis glomerata* (Rakleova et al., 2012).

Expansin-like A1 protein (EXLA1) was more abundant in the medium from LEC cell lines (**Table 1**; **Figure 4**). Expansins play a role in cell wall loosening (Sampedro and Cosgrove, 2005). Particularly, EXLA2 might participate in the initial stage of cell wall regeneration from protoplasts in cotton (Yang et al., 2008). Moreover, EXLA2 was considered a positive regulator of cell elongation in dark grown hypocotyl. However, the changes in the cell wall induced by EXLA are not so drastic as changes induced by expansins (Boron et al., 2015). Precisely controlled cell elongation is essential for proper suspensor formation. HOTHEAD accumulated in the medium from LEC cell lines (**Table 1**; **Figure 4**). The protein HOTHEAD participates in the biosynthesis of long chain α-, ω-dicarboxylic fatty acids and the formation of extracellular matrix (Kurdyukov et al., 2006). HOTHEAD limits the interaction between epidermal cells during pollen or floral development (Krolikowski et al., 2003; Xu et al., 2017).

### 4.2 Signal transduction and secondary metabolism

We found that LysM-domain containing receptor-like kinase (LysM-RLK) accumulated in the medium from HEC cell lines (**Table 1**; **Figure 4**). LysM-RLKs are involved in the perception of signal molecules such as lipo-chitooligosaccharides, chitooligosaccharides, and peptidoglycan produced by microorganisms (Buendia et al., 2018). Endogenous lipo-chitooligosaccharides (Nod factors) promoted early somatic embryo development in Norway spruce (Dyachok et al., 2002). The authors proposed that lipo-chitooligosaccharides suppress the death of embryogenic cells. Another study indicated the crucial role of LysM domain-containing protein for embryo sac development in rice (Zhu et al., 2017). In light of these facts, we propose that LysM-RLK could play a role in the early stages of the SE of black pine.

Ginkbilobin-2 and Gnk domain-containing protein both accumulated in the medium from HEC cell lines (**Table 1**; **Figure 4**). Ginkbilobin is an antifungal protein similar to embryo-abundant proteins from gymnosperms (Wang and Ng, 2000). Ginkbilobin-2 is a full-length secretory protein with a signal peptide (Gao et al., 2016). Its shortened version with cleaved or masked signal peptide bound and visualized the actin cytoskeleton. Shortened ginkbilobin influences actin remodeling and thus may initiate programmed cell death (PCD). Miyakawa et al. (2014) reported carbohydrate-binding properties of Gnk2, which can be essential for its role in seed or embryo development. Based on the similarity of Gnk2 to embryo-abundant proteins, higher abundance in HEC cell lines, and carbohydrate-binding properties, we propose its potential regulatory role in SE.

### 4.3 Protein and nucleic acid metabolism/degradation

Extracellular nuclease S1 and two cysteine proteinases (both papain-like C1 family) accumulated in the medium from LEC cell lines (**Table 1**; **Figure 4**). Both are active in PCD during development or in nutrition acquisition (Solomon et al., 1999; Desai and Shankar, 2003; Parrott et al., 2010; Lesniewicz et al., 2013; Zhao et al., 2013). PCD is a part of normal somatic embryo development and occurs at two time points. The first time point, PCD of pre-embryogenic mass cells, is essential for forming proper somatic embryos (Filonova et al., 2000). Nevertheless, the accumulation of plant S1 nuclease and both cysteine proteinases increased in LEC cell lines, in which the proper formation of somatic embryos does not occur. Based on our results, it is impossible to conclude if the improper regulation of PCD or improper timing of PCD in LEC cell lines affects embryogenic capacity or if the accumulation of nuclease and Cys proteases is a consequence of improper embryo development.

Other interesting proteins, plant basic secretory family protein (BSP) and basic secretory protease, accumulated in the medium from HEC lines (**Table 1**; **Figure 4**). These proteins were induced by exogenous ABA and are putatively involved in the plant defense mechanism against pathogens (Kuwabara et al., 1999). Our results suggest they might play a role in SE as well.

## 5 Conclusion

Our study revealed differences in secretome comparing the cell lines with different embryogenic capacities. The majority of altered proteins are involved in changes of the cell wall composition and remodeling, although some proteins such as peroxidases can have, depending on the developmental context, both loosening and stiffening effects on the cell wall. Both proteomic and microscopy results suggest that cell walls of HEC cell lines, in which cotyledonary embryos are developing, undergo rather stiffening or rigidification and, in contrast, LEC cell lines in which cotyledonary somatic embryos are not developing undergo loosening, softening, or even cell separation. In such scenario, higher activity of peroxidase in HEC might indicate feruloylate cross-linking or lignin deposition, rather than radical-mediated polymer degradation. Although further biochemical and biomechanical experiments are needed to reconcile this, changes that we detected clearly point to the protein-mediated cell wall and starch dynamics likely regulating and defining embryogenic capacity in pine cell cultures. Both peroxidase and α-amylase activities in the culture medium can be used as a marker to distinguish cell lines with HEC and LEC.

## Data availability statement

The datasets presented in this study can be found in online repositories. The names of the repository/repositories and accession number(s) can be found below: https://www.ebi.ac.uk/pride/archive/, PXD040247.

## Author contributions

KK and TS contributed to the conception and design of the study. MP, TS, JB, PB, MD, and KK performed the experiments. MD carried out the statistical analysis. KK wrote the manuscript. All authors contributed to the article and approved the submitted version.
