# Supplementary figures and images for: Secretome analysis revealed that cell wall remodeling and starch catabolism underlie the early stages of somatic embryogenesis in Pinus nigra
Source: Front Plant Sci. 2023 Aug 4;14:1225424. doi: 10.3389/fpls.2023.1225424 (PMC10436561; doi:10.3389/fpls.2023.1225424)

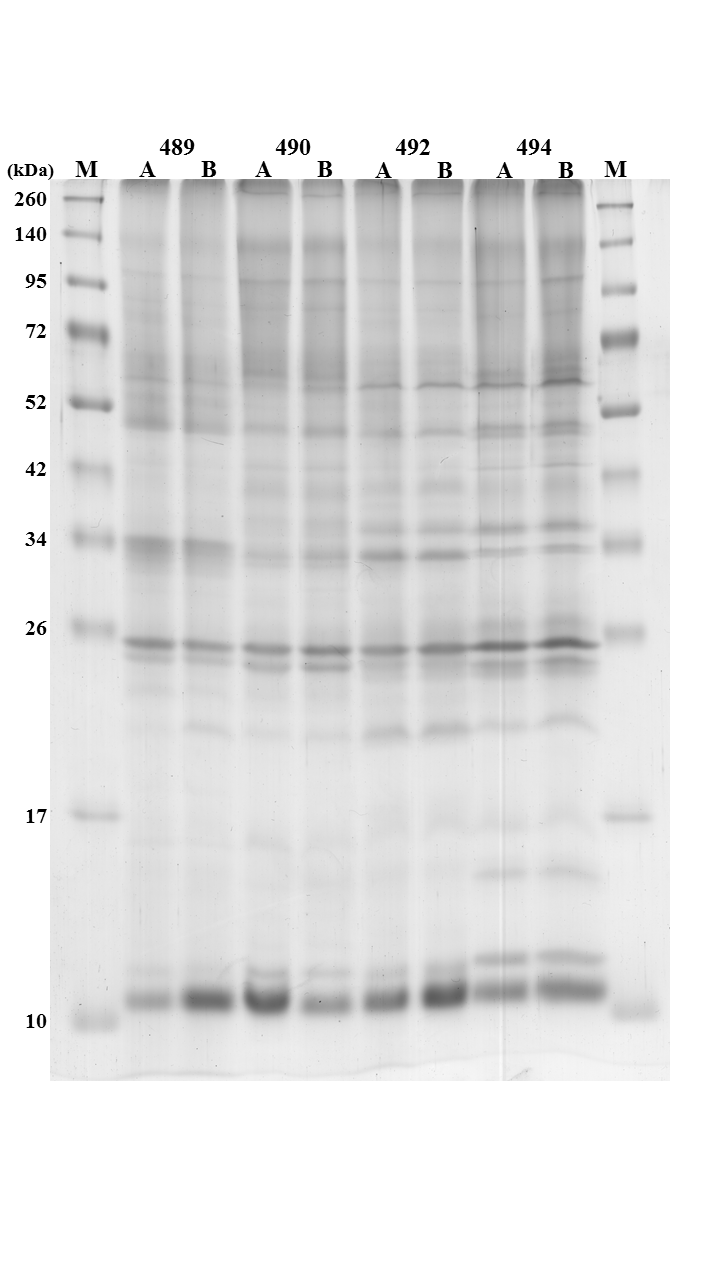

Supplement: Supplementary file 1 [file Image_1.tif]
